# A phase 2 and pharmacological study of sapanisertib in patients with relapsed and/or refractory acute lymphoblastic leukemia

**DOI:** 10.1002/cam4.6701

**Published:** 2023-11-13

**Authors:** Aref Al‐Kali, Ibrahim Aldoss, Pamela J. Atherton, Carrie A. Strand, Bijal Shah, Jonathan Webster, Bhavana Bhatnagar, Karen S. Flatten, Kevin L. Peterson, Paula A. Schneider, Sarah A. Buhrow, Jianping Kong, Joel M. Reid, Alex A. Adjei, Scott H. Kaufmann

**Affiliations:** ^1^ Division of Hematology Mayo Clinic Rochester Minnesota USA; ^2^ Division of Hematology and Hematopoietic Cell Transplantation City of Hope National Medical Center Duarte California USA; ^3^ Department of Biostatics Mayo Clinic Rochester Minnesota USA; ^4^ Division of Hematology Moffitt Cancer Center Tampa Florida USA; ^5^ Division of Hematological Malignancies Johns Hopkins University Baltimore Maryland USA; ^6^ Section of Hematology and Medical Oncology West Virginia University Morgantown West Virginia USA; ^7^ Division of Oncology Research Mayo Clinic Rochester Minnesota USA; ^8^ Division of Medical Oncology Mayo Clinic Rochester Minnesota USA; ^9^ Present address: Tausig Cancer Institute, Cleveland Clinic Cleveland Ohio USA

**Keywords:** acute lymphoblastic leukemia, ALL, mTOR inhibitor, sapanisertib

## Abstract

**Background:**

Despite recent approval of several new agents, relapsed acute lymphoblastic leukemia (ALL) remains challenging to treat. Sapanisertib (MLN0128/TAK‐228) is an oral TORC1/2 inhibitor that exhibited preclinical activity against ALL.

**Methods:**

We conducted a single‐arm multi‐center Phase II study of sapanisertib monotherapy (3 mg orally daily of the milled formulation for 21 days every 28 days) in patients with ALL through the Experimental Therapeutics Clinical Trials Network (NCI‐9775).

**Results:**

Sixteen patients, 15 of whom were previously treated (median 3 prior lines of therapy), were enrolled. Major grade 3–4 non‐hematologic toxicities included mucositis (3 patients) and hyperglycemia (2 patients) as well as hepatic failure, seizures, confusion, pneumonitis, and anorexia (1 patient each). Grade >2 hematological toxicity included leukopenia (3), lymphopenia (2), thrombocytopenia, and neutropenia (1). The best response was stable disease in 2 patients (12.5%), while only 3 patients (19%) were able to proceed to Cycle 2. Pharmacokinetic analysis demonstrated drug exposures similar to those observed in solid tumor patients. Immunoblotting in serially collected samples indicated limited impact of treatment on phosphorylation of mTOR pathway substrates such as 4EBP1, S6, and AKT.

**Conclusion:**

In summary, single‐agent sapanisertib had a good safety profile but limited target inhibition or efficacy in ALL as a single agent. This trial was registered at ClinicalTrials.gov as NCT02484430.

## Graphical abstract

Sapanisertib 3 mg daily was safe for patients with relapsed/refractory acute lymphoblastic leukemia. Its efficacy as single agent is limited.

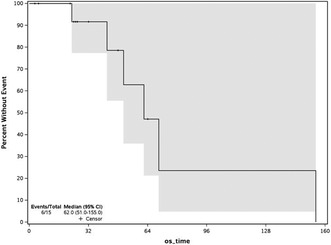

## 1 INTRODUCTION

Acute lymphoblastic leukemia (ALL) is an aggressive hematological malignancy. Despite the use of intensive multi‐agent chemotherapy regimens, relapse is common, particularly in older adults.^1^ Several novel agents were recently approved for relapsed/refractory (r/r) ALL, including blinatumomab (a bispecific T‐cell engager against CD19), tisagenlecleucel and brexucabtagene autoleucel (chimeric antigen‐receptor T‐cells against CD19), and inotuzumab ozogamicin (antibody‐drug conjugate against CD22). Nonetheless, responses to these agents can be short‐lived, especially if allogeneic hematopoietic cell transplantation is not available.^2^, ^3^, ^4^, ^5^, ^6^, ^7^ Many other classes of medications are currently undergoing testing in r/r ALL, including monoclonal antibodies, demethylating agents, cell cycle inhibitors, BCL2 family inhibitors, and mechanistic targets of rapamycin (mTOR) inhibitors.^8^, ^9^, ^10^, ^11^

The rationale for targeting mTOR in ALL comes from previous studies of the effects of mTOR inhibitors on lymphoid cells. The natural product sirolimus (rapamycin) and its derivatives, which were the first identified mTOR inhibitors, not only modulate proliferation of normal T cells,^12^, ^13^, ^14^ contributing to beneficial immunosuppressive effects,^15^, ^16^ but also impact survival of malignant lymphoid cells.^17^, ^18^, ^19^, ^20^, ^21^ However, rapamycin and its derivatives do not completely inhibit mTOR signaling. The mTOR protein is a component of two distinct signaling complexes, TORC1, which phosphorylates p70 S6 kinase and 4EBP1 to regulate protein synthesis, and TORC2, which phosphorylates AKT on Ser^473^ to activate anti‐apoptotic signaling.^22^ This anti‐apoptotic TORC2 signaling has been implicated in resistance to rapamycin analogs, which inhibit TORC1 but usually not TORC2.^22^, ^23^, ^24^, ^25^ To overcome this potential mechanism of resistance, dual TORC1/TORC2 inhibitors^26^, ^27^ were evaluated and found to be more cytotoxic than rapamycin in primary samples of a variety of lymphoid neoplasms exposed ex vivo.^28^, ^29^ Importantly, across a series of lymphoid neoplasms, ALL was observed to be the most sensitive to the dual TORC1/TORC2 inhibitor OSI‐027.^28^

Sapanisertib (TAK‐228, MLN0128) is an orally bioavailable dual TORC1/TORC2 inhibitor that inhibits proliferation of a wide range of tumor cell lines.^30^ Like the earlier TORC1/TORC2 inhibitor OSI‐027, sapanisertib exhibited greater cytotoxic effects than rapamycin in multiple human ALL lines and in clinical ALL samples treated ex vivo.^29^ Sapanisertib also inhibited growth of a syngeneic mouse model of BCR‐ABL‐positive ALL^30^ and several human ALL cell line xenografts in vivo.^29^ In humans, sapanisertib was tested in patients with advanced solid malignancies and advanced lymphoid neoplasms. In the INK128‐001 study, the maximum tolerated dose (MTD) of the original drug formulation was 6 mg/day for daily dosing. GI disturbances and hyperglycemia were the most frequent adverse events.^31^ In the INK128‐002 study in advanced multiple myeloma, the MTD was also 6 mg orally daily, with GI disturbances and hyperglycemia again being the most frequent adverse events.^32^ Based on promising results in *NRF2*‐mutated squamous cell carcinoma of the lung, sapanisertib was recently granted fast‐track designation by the Food and Drug Administration.

In light of these preclinical and clinical results, we conducted an open–label, multi‐institution Phase 2 study of single‐agent sapanisertib in patients with relapsed/refractory ALL that included measurement of plasma sapanisertib levels as well as serial analysis of bone marrow samples to assess target pathway inhibition.

## 2 METHODS

### 2.1 Patient selection

Adult patients with B‐ or T‐cell acute lymphoblastic leukemia who failed (relapsed or refractory) two prior treatments but had good performance status (<3) and organ function were initially enrolled. The protocol was later amended due to low accrual to allow any number of prior lines of therapy and patients who were newly diagnosed but unfit for standard induction therapy. Active leukemia (≥10% blasts in the bone marrow) was mandated to assess response to therapy. Prior hematopoietic cell transplant or the presence of Philadelphia chromosome‐positive (as long as they failed all available therapy against Philadelphia chromosome) disease were allowed. Fasting blood glucose <130 mg/dL was mandated because of the known hyperglycemia associated with mTOR inhibitors. Concurrent proton pump inhibitors and prolonged QT interval (corrected QT >480 milliseconds) were not allowed.

### 2.2 Treatments

Sapanisertib was administered in the milled formulation at a dose of 3 mg orally daily for 21 days every 28 days (Figure S1). This dose was chosen in collaboration with investigators at Takeda and CTEP based on a known MTD of 6 mg orally daily in early studies and demonstrated greater bioavailability of the milled formulation used in the present study. Responders could continue on the same dose or proceed to allogeneic hematopoietic cell transplant if available. Non‐responders (but tolerating drug) could increase their dose to 4 mg orally for 21 days every 28 days (dose level + 1) starting in Cycle 3 and 4 mg orally continuously (dose level + 2) starting in Cycle 5 (if they tolerated dose level + 1). Dose reduction was recommended for toxicity (dose level − 1: 2 mg daily for 21 days every 28 days; dose level − 2: 2 mg daily for 5 days/week for 3 weeks every 4 weeks). Treatment was to be discontinued if a patient did not respond after a total of six cycles. Intrathecal chemotherapy was allowed per institutional standard policies. In‐home blood glucose monitoring was performed daily to monitor blood sugar (a glucometer was given to every patient on the study). Discontinuation of therapy was recommended for disease progression, severe toxicity, lack of response, proceeding to hematopoietic cell transplant, or prolonged delay of the subsequent cycle (>1 month delay for therapy‐related toxicity or >2 months delay for unrelated toxicity).

### 2.3 Response assessment

Evaluable patients for response include all patients who had measurable disease present at baseline, began treatment, and had their disease re‐evaluated. Response was assessed by bone marrow aspirates and biopsies performed every 2 months (mandated at the end of Cycle 2, Cycle 4, and Cycle 6). The best responses were captured as overall responses at any time point. Response was evaluated based on modified response criteria by the International Working Group (IWG) recommendations for acute myeloid leukemia (Table S1).^33^

### 2.4 Statistics

This was a phase II trial designed using a Simon design with an interim analysis to evaluate efficacy. Efficacy (success) was defined as a complete response (CR or CR incomplete (i)). All patients meeting the eligibility criteria who signed a consent form and began treatment were deemed evaluable for toxicity. Similar studies showed a CR rate ranging from 20 to 47% in this patient population.^34^, ^35^, ^36^ Thus, this regimen was to be considered ineffective if the success rate was 10% or less (null hypothesis), while the smallest success rate that would warrant subsequent studies was 30% (i.e., alternate research hypothesis). With a maximum of 26 evaluable patients, this trial had 85% power to detect a CR/CRi rate of 30% or greater (vs. 10% or less) with a one‐sided Type I error of 0.09. This trial was designed with a futility analysis after enrollment of 11 patients; if there were one or fewer successes observed in these first 11 patients, the regimen would be considered ineffective, and the study terminated. If two or more successes were observed, the enrollment would continue to a total of 26 evaluable patients. In the final analysis, if five or more of these 26 evaluable patients had achieved a CR or CRi, this would be considered sufficient evidence of promising activity in this patient population. Otherwise, if four or fewer CR/CRi events were observed, this regimen would not warrant further evaluation in this population.

The primary endpoint was the proportion of successes, which was estimated by the number of successes divided by the total number of evaluable patients. Secondary endpoints of duration of response (CR or CRi) and overall survival (OS) were evaluated using Kaplan–Meier techniques; the frequency of proceeding to allogeneic stem cell transplant after achieving response and adverse events (AEs), as evaluated using reported the NCI Common Terminology Criteria for Adverse Events (CTCAE) version 4.0, were presented using descriptive statistics.

### 2.5 Pharmacokinetics

Blood samples were obtained on Days 1, 2, and 8 of Cycle 1. TAK‐288 plasma concentrations were measured using liquid chromatography‐mass spectroscopy. Plasma concentration‐time data were analyzed by standard methods using WinNonlin. Pearson's correlation coefficient (*r*) was used to assess correlation of group variables. Statistical significance was defined as *p* < 0.05. Additional details are contained in the Supplementary Method: Data S1.

### 2.6 Impact of treatment on phosphorylation of substrates in the mTOR pathway

Bone marrow aspirates harvested prior to treatment and again prior to drug administration on Day 8 ± 2 of treatment from the same patients were shipped overnight to a central laboratory, where marrow mononuclear cells isolated on Ficoll–Hypaque gradients were lysed under denaturing conditions that preserve protein phosphorylations. Paired whole cell lysates were subjected to SDS‐polyacrylamide gel electrophoresis and immunoblotting as described in the Supplementary Method: Data S1.

## 3 RESULTS

Sixteen patients were enrolled between December 2016 and December 2018, at six centers. Their median age was 52.4 years, with 56% being males (Table 1). All patients but one had r/r ALL, with B‐cell lineage being the most frequent subtype (9 patients B‐cell, 4 T‐cell, 3 unknown). The majority of patients were heavily treated, with a median of 3.5 prior treatments (range, 1–7). Thirty‐eight percent had prior allogeneic hematopoietic cell transplantation.

**TABLE 1:** Patient baseline demographics.

	Total (*N* = 16)
Age	
N	16
Mean (SD)	52.4 (19.9)
Median	45.5
Q1, Q3	36.0, 75.0
Sex	
Female	7 (43.8%)
Male	9 (56.3%)
Race	
American Indian or Alaska Native	1 (6.3%)
Asian	2 (12.5%)
White	11 (68.8%)
Unknown	2 (12.6%)
Ethnicity	
Hispanic or Latino	4 (25.0%)
Not Hispanic or Latino	11 (68.8%)
Unknown	1 (6.3%)
PS	
1	11 (68.8%)
2	5 (31.3%)
ALL lineage	
B‐cell	9 (31%)
T‐cell	4 (25%)
Unknown	3 (44%)
Prior treatment	
Bone marrow transplantation	5 (31.3%)
Chemotherapy, NOS	3 (18.8%)
Chemotherapy, multiple agents systemic	11 (68.8%)
Immunotherapy	4 (25.0%)
Limited radiation therapy	1 (6.3%)
Prior therapy (NOS)	3 (18.8%)
Radiation therapy (NOS)	1 (6.3%)
Single agent systemic chemotherapy	12 (75.0%)
Surgery	15 (93.8%)

One patient was ineligible and did not receive treatment. Although 15 patients started Cycle 1 of treatment, 4 received <40% of the expected Cycle 1 sapanisertib dosing and 11 received >40% of the expected Cycle 1 dosing. Three of these 15 patients received 4 or fewer days of sapanisertib. The median duration of sapanisertib treatment was 16 days (range 0–64), and only 3 patients received any Cycle 2 of treatment. Reasons patients went off study after receiving <28 days of sapanisertib include disease progression (*N* = 6), consent withdrawal (*N* = 2), transitioning to hospice care (*N* = 3), and quitting due to side effects (*N* = 1). Patients who received Cycle 2 of treatment went off treatment due to disease progression (*N* = 2) and no response to treatment (*N* = 1).

### 3.1 Safety

Safety was evaluated in 15 patients. All 15 patients had at least 1 grade 3 AE, while 9 patients had a grade 3 or higher AE related to the study drug. Grade 3–4 treatment‐related non‐hematological AEs included mucositis (3), hyperglycemia (2), anorexia (1), confusion (1), delirium (1), abdominal pain (1), bilirubin increase (1), febrile neutropenia (1), fibrinogen decrease (1), thoracic disease (1), and pneumonitis (1). Two patients suffered G5 death shortly after coming off the protocol due to disease progression. Grade 3–4 hematological AEs, at least possibly related to treatment, included anemia (4), lymphopenia (2), leukopenia (2), leukocytosis (1), thrombocytopenia (4), and neutropenia (1) (Table 2, Table S2). A comparison of toxicity based on cell lineage (B‐ vs. T‐cell) showed more frequent related (grade >2) hematological adverse events (thrombocytopenia) in T‐cell cases, whereas B‐cell cases had more frequent (grade >2) non‐hematological events (mucositis and hyperglycemia) (Table S3).

**TABLE 2:** Treatment‐related adverse events on study.

Listing of grade 1+ adverse events max grade per patient per event regardless of attribution number of evaluable patients arm: *A* = 15					
	Grade of adverse event				
	1	2	3	4	5
	*N* (%)	*N* (%)	*N* (%)	*N* (%)	*N* (%)
Hematologic adverse events					
Blood/Bone marrow					
Anemia	0 (0)	0 (0)	4 (27)	0 (0)	0 (0)
Leukocytosis	0 (0)	0 (0)	1 (7)	0 (0)	0 (0)
Lymphocyte count decreased	0 (0)	0 (0)	2 (13)	0 (0)	0 (0)
Neutrophil count decreased	0 (0)	0 (0)	0 (0)	1 (7)	0 (0)
Platelet count decreased	0 (0)	0 (0)	1 (7)	3 (20)	0 (0)
White blood cell decreased	0 (0)	0 (0)	0 (0)	2 (13)	0 (0)
Non‐hematologic adverse events					
Blood and lymphatic					
Febrile neutropenia	0 (0)	0 (0)	1 (7)	0 (0)	0 (0)
Gastrointestinal disorders					
Abdominal pain	0 (0)	0 (0)	1 (7)	0 (0)	0 (0)
Anal mucositis	0 (0)	1 (7)	0 (0)	0 (0)	0 (0)
Constipation	1 (7)	0 (0)	0 (0)	0 (0)	0 (0)
Diarrhea	1 (7)	0 (0)	0 (0)	0 (0)	0 (0)
Mucositis oral	0 (0)	0 (0)	3 (20)	0 (0)	0 (0)
Oral pain	1 (7)	0 (0)	0 (0)	0 (0)	0 (0)
Vomiting	1 (7)	0 (0)	0 (0)	0 (0)	0 (0)
General and administration					
Edema limbs	1 (7)	0 (0)	0 (0)	0 (0)	0 (0)
Fatigue	1 (7)	0 (0)	1 (7)	0 (0)	0 (0)
Investigations					
Aspartate aminotransferase increased	1 (7)	0 (0)	0 (0)	0 (0)	0 (0)
Blood bilirubin increased	1 (7)	0 (0)	1 (7)	0 (0)	0 (0)
Fibrinogen decreased	0 (0)	0 (0)	1 (7)	0 (0)	0 (0)
Metabolic					
Anorexia	0 (0)	1 (7)	1 (7)	0 (0)	0 (0)
Hyperglycemia	0 (0)	0 (0)	2 (13)	0 (0)	0 (0)
Hypertriglyceridemia	1 (7)	0 (0)	0 (0)	0 (0)	0 (0)
Nervous system disorders					
Dysgeusia	1 (7)	0 (0)	0 (0)	0 (0)	0 (0)
Psychiatric disorders					
Anxiety	1 (7)	0 (0)	0 (0)	0 (0)	0 (0)
Confusion	0 (0)	0 (0)	1 (7)	0 (0)	0 (0)
Reproductive system					
Vaginal inflammation	0 (0)	1 (7)	0 (0)	0 (0)	0 (0)
Respiratory					
Cough	1 (7)	0 (0)	0 (0)	0 (0)	0 (0)
Pharyngolaryngeal pain	0 (0)	1 (7)	0 (0)	0 (0)	0 (0)
Pneumonitis	0 (0)	0 (0)	1 (7)	0 (0)	0 (0)
Resp, thoracic, mediastinal	0 (0)	0 (0)	0 (0)	1 (7)	0 (0)
Skin and subcutaneous					
Pruritus	1 (7)	0 (0)	0 (0)	0 (0)	0 (0)
Others					
Bloating	1 (7)	0 (0)	0 (0)	0 (0)	0 (0)
Delirium	0 (0)	0 (0)	1 (7)	0 (0)	0 (0)
Fall	0 (0)	1 (7)	0 (0)	0 (0)	0 (0)
Neoplasms	0 (0)	0 (0)	1 (7)	0 (0)	0 (0)
Paresthesia	1 (7)	0 (0)	0 (0)	0 (0)	0 (0)
Wheezing	0 (0)	1 (7)	0 (0)	0 (0)	0 (0)

### 3.2 Efficacy and survival

**FIGURE 1:**
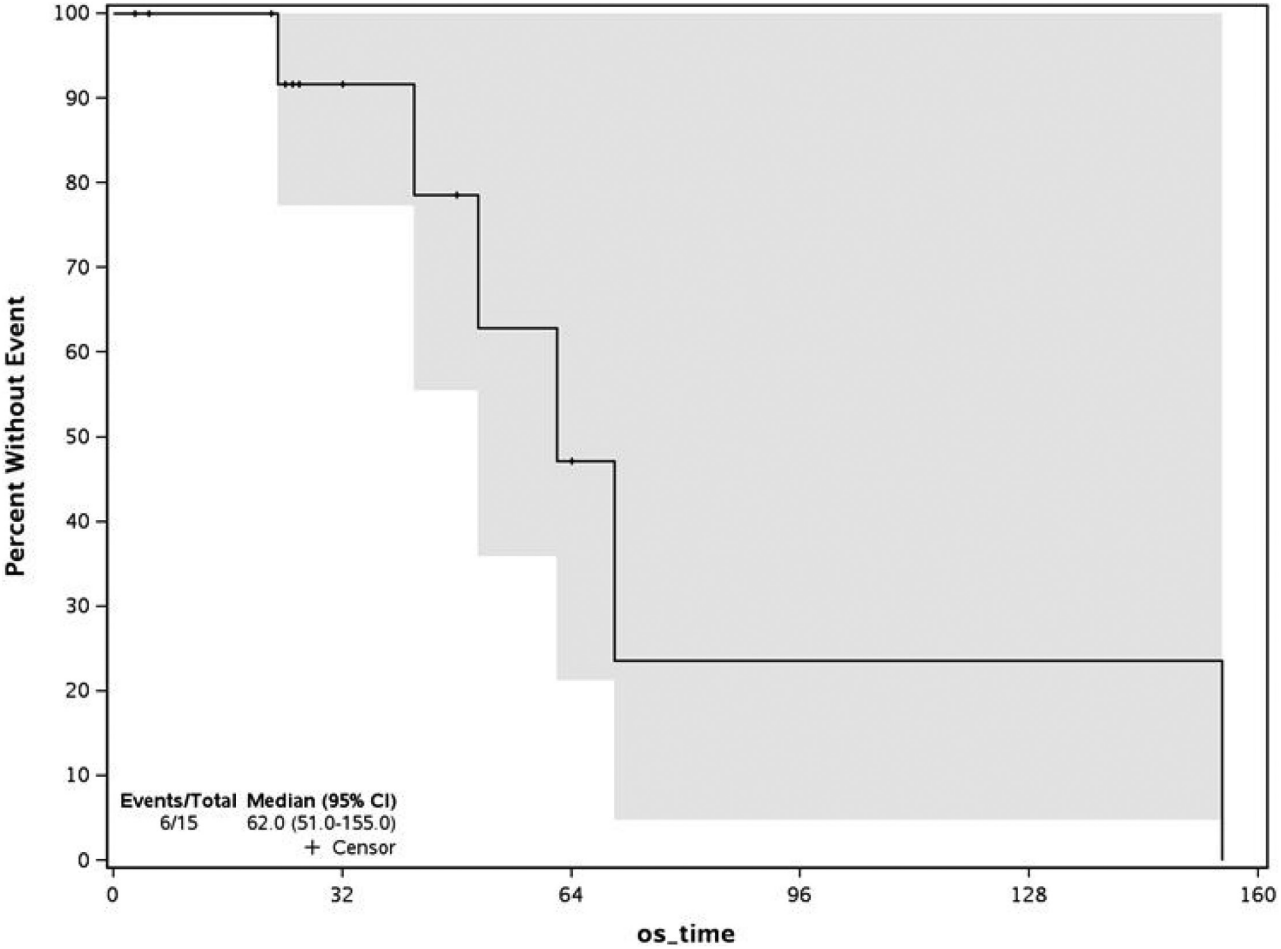
Overall survival for relapsed/refractory acute lymphoblastic leukemia patients treated with oral sapanisertib.

No patient achieved a CR or CRi. The best response was stable disease in 3 patients (19%). 6 (38%) patients had disease progression, whereas 7 (44%) patients were not evaluable because of early discontinuation of sapanisertib. Median follow‐up time for response was 22 days (range 3–64 days). Median survival was 62 days (51.0‐NA 95% CI), and all 6 patients with disease progression were deceased by day 155, reflecting the heavily pretreated cohort (Figure 1). Due to lack of clinical efficacy, the protocol was stopped at interim analysis due to futility.

### 3.3 Pharmacokinetics

A total of 14 patients were evaluated for PK during Cycle 1. All patients were treated with 3 mg daily of sapanisertib. Descriptive statistics of the mean PK parameters on Cycle1 Day 1 and Day 8 are summarized in Table 3. During the first dose in Cycle 1, the mean sapanisertib peak concentration (*C*_max_) of 26.1 ± 8.0 ng/mL was achieved 2 h (range, 0.5–4.1 h) after drug administration on Day 1. The mean elimination half‐life, AUC_0–24h_ and clearance values of sapanisertib on Day 1 were 8.2 ± 4.9 h, 213 ± 143 hr*ng/mL, and 22.0 ± 14.2 L/hr, respectively. Modest accumulation of sapanisertib was observed comparing Day 8 versus Day 1 AUC_all_, with a mean accumulation ratio of 1.4. The mean *C*_max_ of 34.4 ± 20.2 ng/mL was achieved 1.2 h (range, 0.5–2.9 h) after sapanisertib administration on Day 8. The mean half‐life, AUC_0–24h_, and clearance values were 24.3 ± 42.5 h, 361 ± 337 hr*ng/mL, and 23.3 ± 23.8 L/hr, respectively. There was no significant correlation between systemic sapanisertib clearance and sex, age, or total body weight (*p* > 0.05).

**TABLE 3:** Descriptive statistics of the mean sapanisertib PK parameters in Cycle 1 Day 1 (Day 1) and Cycle 1 Day 8 (Day 8).

	Day 1		Day 8	
	Mean ± SD	Median (range)	Mean ± SD	Median (range)
*C*_max_ (ng/mL)	26.1 ± 8.0	26.5 (14.9–41.8)	34.4 ± 20.9	29.4 (3.6–68.8)
*T*_max_ (hr)	1.7 ± 1.1	2.0 (0.5–2.8)	1.5 ± 0.7	1.2 (0.5–2.9)
Half‐life (hr)	8.2 ± 4.9	7.1 (2.1–16.0)	24.3 ± 42.5	11.3 (2.4–144)
AUC0–24 h (hr*ng/mL)	213 ± 143	213 (61–516)	361 ± 337	182 (36.3–947)
CL (L/hr)	22.0 ± 14.2	22.0 (5.8–49.2)	23.3 ± 23.8	16.4 (3.2–82.6)

Further exploratory analysis was undertaken to evaluate the relationship between sapanisertib systemic clearance and toxicity. AEs were classified as hematological and non‐hematological. The subjects with hematologic AEs experienced anemia, neutropenia, febrile neutropenia, thrombocytopenia, lymphocytopenia, or leukocytosis. Six of 11 patients (54.5%) had at least one grade ≥3 non‐hematologic AE: fatigue, anorexia, delirium, hyperglycemia, high blood bilirubin level, oral mucositis, pneumonitis, and others. No significant correlation was found between systemic sapanisertib exposure and hematologic toxicity after drug administration on either Cycle 1 Day 1 or Day 8 (Table 4). However, patients displayed a correlation between systemic sapanisertib exposure and non‐hematologic AEs on Cycle 1 Day 1 (*p* = 0.03) and a trend toward significance on Day 8 (*p* = 0.08).

**TABLE 4:** The correlation analysis of sapanisertib clearance and toxicity.

Toxicity	Pearson correlation		*p* Value	
	Day 1	Day 8	Day 1	Day 8
Hematologic	−0.2575	0.05028	0.44 (NS)	0.8833 (NS)
Non‐hematologic	−0.647	−0.5028	0.0314 (Yes)	0.075 (NS)

### 3.4 Impact of sapanisertib on mTOR‐induced protein phosphorylations

Pretreatment samples were available from 14 patients enrolled in the study. Immunoblotting revealed that baseline levels of phospho‐Thr^35^, ^46^‐4EBP1 (a direct substrate of mTORC1^37^), phospho‐Ser^240,244‐^S6 (substrate of S6K1 downstream of the mTORC1) and phospho‐Ser^473^‐AKT (a direct substrate of mTORC2) were detectable in most samples, indicating the presence of mTOR activity, albeit at widely varying levels (Figure S2).

**FIGURE 2:**
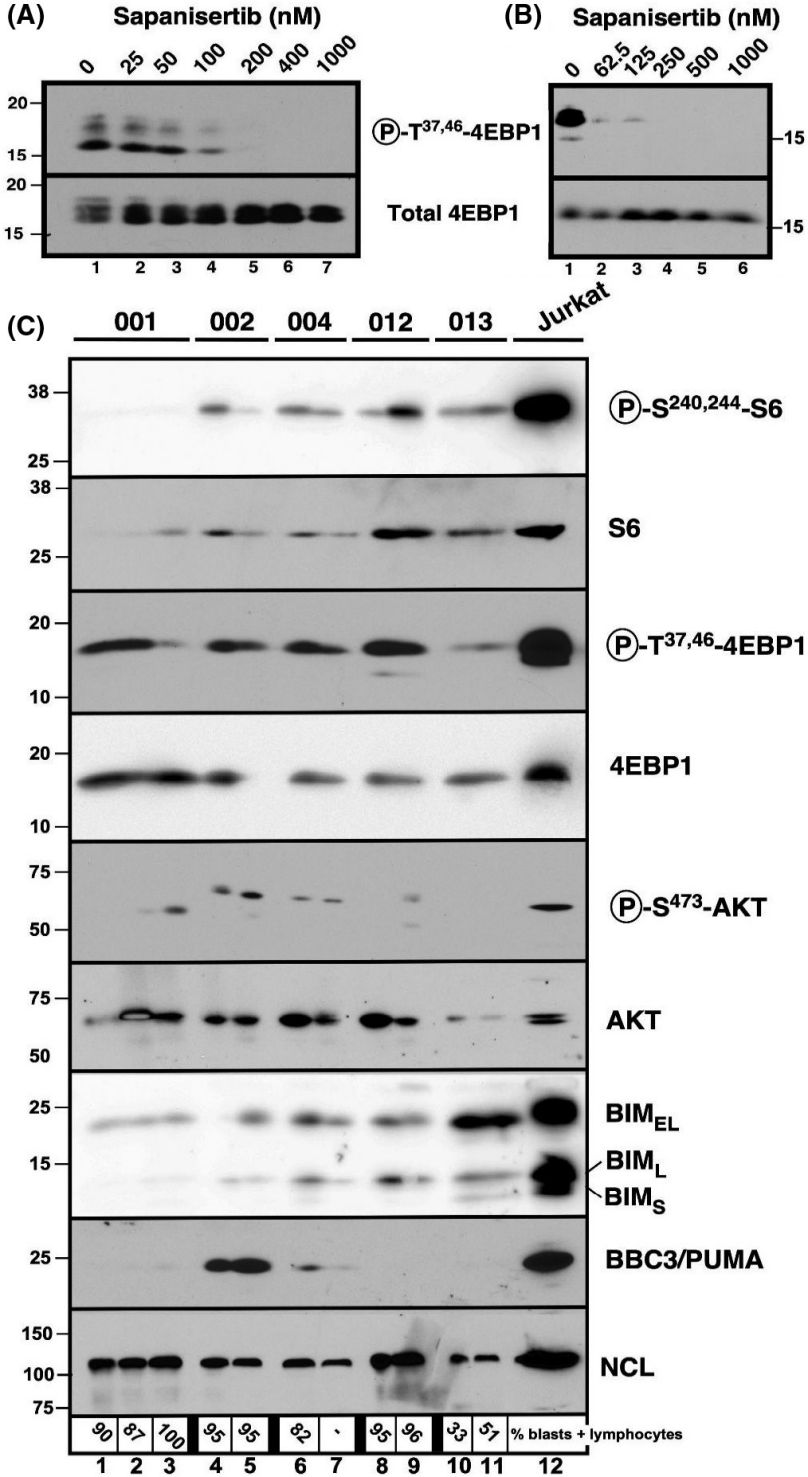
Effect of MLN0128 concentrations on mTOR‐associated phosphorylation in vitro, ex vivo, and in the context of the present trial. (A) Log phase Jurkat cells were treated for 24 h with diluent (0.1% DMSO, lane 1) or sapanisertib at the indicated concentration, lysed, and subjected to immunoblotting for phospho‐Thr^36^, ^45^‐4EBP1 or total 4EBP1. (B) Replicate aliquots isolated from a pretreatment ALL specimen from a patient not enrolled on this trial were treated for 48 h with 0.1% DMSO or the indicated concentration of sapanisertib in the presence of 5 μM Q‐VD‐OPh, washed, and subjected to SDS‐PAGE followed by blotting for the indicated antigen. (C) Samples harvested from the indicated patients prior to treatment (lanes 1, 4, 6, 8, and 10), on Day 8 prior to therapy (lanes 2, 5, 7, 9, and 11), or at the time of removal from study after 6 weeks (lane 3) were subjected to SDS‐PAGE and blotting for the indicated antigens. Jurkat cells (lane 12) were included in the blot because they were known to be positive for all analytes.^29^ The housekeeping protein nucleolin (NCL) served as a loading control.

In further analysis, serial samples harvested from five patients prior to therapy and again on Day 8 before drug administration (at the time of daily trough concentrations) were examined for evidence of sapanisertib‐induced changes in mTOR‐mediated phosphorylations. To provide a basis for comparison, the Jurkat ALL cell line and an ALL sample were exposed to sapanisertib ex vivo. These studies indicated that sapanisertib diminished phosphorylation of phospho‐Thr^35^, ^46^‐4EBP1, a direct mTORC1 substrate, at 62.5–200 nM sapanisertib without any change in total 4EBP1 ex vivo (Figure 2A,B). In contrast, every change in phospho‐4EBP1, phospho‐S6, or phospho‐AKT in trial samples in situ was accompanied by a corresponding change in the total 4EBP1, S6, or AKT (Figure 2C). While we cannot completely rule out the possibility that these changes reflect an impact of mTOR inhibition of transcription,^38^ the present results provide little evidence that mTOR‐mediated phosphorylation has been disrupted. Consistent with this conclusion, induction of BIM_EL_ and PUMA, the effectors of mTORC1/mTORC2 dual inhibitors,^29^ was minimal in one pair of samples (Patient 2) and absent from the other four pairs (Figure 2C).

## 4 DISCUSSION

ALL in adults continues to be a challenging disease with a high relapse rate despite intensive treatment with multi‐agent chemotherapy. The addition of immunotherapy (rituximab, ofatumumab) may help in decreasing relapse and improving outcomes when compared to historical data (especially in patients younger than 60 years old).^39^, ^40^ However, once relapse occurs, allogeneic hematopoietic cell transplantation becomes the only potentially curative option. Several newer agents were recently approved for r/r ALL as monotherapy, including bispecific T‐cell engagers, second‐generation antibody‐drug conjugates, and chimeric antigen‐receptor T‐cells (CAR‐T). Two CAR‐T products are now available in the US for treating r/r ALL; tisagenlecleucel was approved in 2017 (age < 26 years old) and brexucabtagene autoleucel in 2021 for all r/r ALL (regardless of age) patients. Such patients should have CD19+ leukemia (applicable in B‐cell lineage), have controllable disease during product manufacturing, be able to tolerate severe toxicities (like cytokine release syndrome and neurotoxicity), and their care is mainly available at tertiary centers. Because many of these treatments are available for r/r ALL, studies of new investigational drugs (especially as monotherapy) must enroll heavily pretreated patients, often with the most resistant disease. In our study, the median lines of prior therapy were 3, with some patients having nine prior treatments. Such heavily pretreated and refractory cases ended up withdrawing from study prematurely, with only 3 (18.8%) completing one full cycle. In contrast, one previously untreated patient (unfit for chemotherapy) had myelosuppression (pancytopenia), indicating an on‐target effect in the hematopoietic cells. Unfortunately, that patient developed mucositis and discontinued the study, precluding knowing whether a response would be attained or not. The study provides a proof‐of‐concept that targeting the mTOR pathway is possible in ALL patients, although in r/r ALL, a backbone of other agents (corticosteroids or other agents) may be needed.

In terms of safety, no G5 toxicity was encountered. Hyperglycemia was seen in 4 (25%) of patients at a median time of 6 days and was managed with oral hypoglycemics (2 patients with G2 and 2 patients with G3). Other toxicities included mucositis in 3 patients. It is unknown whether the mucositis was related to disease itself, prior chemotherapy or direct drug toxicity. One could argue that safety information about sapanisertib in ALL is limited because most patients had 1 cycle or less of treatment, and long‐term side effects could not be assessed in this population.

Sapanisertib clearly failed to demonstrate efficacy as monotherapy in this study population. In particular, no patient achieved a CR or CRi. Instead, the best response was stable disease in 2 patients (12.5%). This does not compare favorably with other studies of mTOR inhibitors in ALL. Everolimus, an mTORC1 inhibitor, was combined with Hyper‐CVAD in a Phase I/II study in patients with r/r ALL by Daver et al.^41^ An overall response rate of 33% was observed. Six of 11 patients who had received only one prior line of therapy achieved a CR or CRi. Half of the heavily pretreated T‐ALL patients also achieved a response. The dose‐limiting toxicity in this trial was G3 mucositis. In a phase I study by Rheingold et al.,^42^ temsirolimus (another mTORC1 inhibitor) was added to a backbone of intensive chemotherapy (Children Oncology Group, ADVL1114) in pediatric patients with r/r ALL in second relapse or greater. Seven of 15 (47%) patients achieved remission, with three patients (20%) having minimal residual disease <0.01%. However, toxicity was seen in all dose levels. DLTs included mucositis, hypertriglyceridemia, elevated liver enzymes, hypertension, and sepsis. In a subsequent study, r/r pediatric ALL patients were treated with cyclophosphamide, etoposide, and temsirolimus.^43^ Among 15 patients, one DLT (grade 4 pleural and pericardial effusion) was observed. Other toxicities included elevated alanine aminotransferase and mucositis. Despite extensive prior therapy, with eight patients in their second relapse and six in their third or higher, an overall response rate of 47% (26% CR or CRi) was observed. Finally, Place et al. combined everolimus with multi‐agent chemotherapy (including pegaspargase) in children and adolescents with ALL at first relapse after 18 or more months in first CR.^44^ Nineteen of 22 patients (86%) obtained remission (as CR2) with this regimen. DLT was elevated liver enzymes in one patient. Two out of twelve (17%) patients at dose level 3 had meningitis. It is noteworthy that none of these trials examined mTORC1 inhibitors as monotherapy. Thus, it remains to be determined whether mTORC1 inhibitors have monotherapy activity in ALL and how much mTORC1 inhibition contributes to the efficacy of the backbone chemotherapies in these studies.

The laboratory studies conducted in conjunction with the present trial permit at least a partial analysis of the lack of efficacy of sapanisertib monotherapy. The sapanisertib pharmacokinetics observed in this study were similar to those found in other trials.^31^, ^32^, ^45^, ^46^ Peak concentrations of 26.1 ng/mL were achieved 1.7 h after administration of the 3 mg dose on Day 1. Little drug accumulation was observed after daily doses, with a peak concentration of 34.4 ng/mL achieved 1.5 h after the Day 8 dose. The modest accumulation was consistent with a half‐life of 8 h. A borderline statistically significant correlation was identified between non‐hematologic toxicity and sapanisertib clearance.

Analysis of paired samples harvested prior to therapy and on Day 8 failed to convincingly demonstrate inhibition of mTOR‐induced phosphorylations (Figure 2C). This is in contrast to studies of mTORC1 inhibitor‐containing regimens in r/r ALL, which demonstrated inhibition of RPS6^41^, ^43^ or 4EBP1 phosphorylation.^42^ The negative results in the present trial suggest that sapanisertib levels achieved in bone marrow were unable to inhibit mTORC1 and mTORC2 signaling. In this context, it is noteworthy that peak plasma concentrations of 26 ng/mL (84 nM) were achieved on Day 1. While this would be in the range of concentrations required to inhibit mTOR‐induced phosphorylations in ALL lines and clinical samples (Figure 2A,B), it is important to emphasize that sustained concentrations in this range for 48–72 h are required for cell killing.^29^ The pharmacokinetic analysis performed in conjunction with this trial, however, indicates a terminal serum half‐life of 8 h and median trough concentrations of 1.9 ng/mL (range, 0–41.6 ng/mL) after 7 days of dosing. Accordingly, plasma drug concentrations are below the threshold required for target inhibition and killing for the majority of each 24‐h period. Thus, if sapanisertib is to be tested again in ALL, it will be important to administer doses that achieve higher plasma concentrations and, in view of results with mTORC1 inhibitors, to consider administering TORC1/2 inhibitors in conjunction with additional agents. Because preclinical models indicate continued growth of lymphoid xenografts for up to a week after initiation of effective TORC1/2 inhibitor therapy,^28^ it might also be important to continue suppressive therapy such as glucocorticoids or hydroxyurea for an extended period of time after initiating TORC1/2 inhibitor therapy, which was not done in the present trial.

In conclusion, single‐agent sapanisertib exhibited the expected toxicity profile, including hyperglycemia and mucositis. As a single agent, it induced myelosuppression in several cases but did not display objective activity against ALL on the dose and schedule administered. Further work using an mTOR inhibitor that is more selectively targeted toward lymphoid cells or a TORC1/2 inhibitor as part of combination therapy might be warranted in this population with unmet needs.

## AUTHOR CONTRIBUTIONS

**Aref Al‐Kali:** Conceptualization (lead); formal analysis (lead); funding acquisition (lead); investigation (lead); methodology (lead); project administration (lead); writing – original draft (lead); writing – review and editing (equal). **Ibrahim Aldoss:** Formal analysis (supporting); investigation (equal); project administration (equal); writing – review and editing (equal). **Pamela J. Atherton:** Data curation (lead); formal analysis (lead); investigation (equal); methodology (lead); writing – original draft (lead); writing – review and editing (equal). **Carrie A. Strand:** Conceptualization (equal); data curation (equal); formal analysis (equal); writing – original draft (equal); writing – review and editing (equal). **Bijal Shah:** Investigation (equal); project administration (equal); writing – original draft (equal); writing – review and editing (equal). **Jonathan Webster:** Investigation (equal); project administration (equal); writing – review and editing (equal). **Bhavana Bhatnagar:** Investigation (equal); project administration (equal); writing – review and editing (equal). **Karen S. Flatten:** Data curation (equal); writing – review and editing (equal); writing – review and editing (equal). **Kevin L. Peterson:** Data curation (equal); writing – review and editing (equal). **Paula A. Schneider:** Data curation (equal); formal analysis (equal); writing – review and editing (equal). **Sarah A. Buhrow:** Data curation (equal); formal analysis (equal); writing – review and editing (equal). **Jianping Kong:** Data curation (equal); formal analysis (equal); writing – review and editing (equal). **Joel M. Reid:** Conceptualization (lead); data curation (lead); formal analysis (lead); methodology (lead); writing – original draft (lead); writing – review and editing (lead). **Alex A. Adjei:** Resources (lead); writing – review and editing (equal). **Scott H. Kaufmann:** Conceptualization (lead); data curation (lead); formal analysis (lead); funding acquisition (lead); investigation (lead); methodology (lead); project administration (lead); writing – original draft (lead); writing – review and editing (lead).

## FUNDING INFORMATION

This study was supported by an NCI grant for Experimental Therapeutics – Clinical Trials Network with phase 1 emphasis (UM1 CA186686).

## CONFLICT OF INTEREST STATEMENT

Authors declare no relevant conflict of interest.

## ETHICS STATEMENT

This trial was conducted under the approval of central IRB, where the Adult NCI IRB was the IRB of Record for this NCI trial (9775), prior to commencing this study. Additionally the IRB approval was renewed on a yearly basis. All patients on this protocol consented prior to commencing any research activities and while on study. All local sites had this study reviewed and acknowledged by the local IRB, where the Adult NCI IRB was the IRB of record. The lead institution (Mayo Clinic) had its local IRB review and approve this study (IRB‐008314).

## PRIOR PUBLICATIONS

Part of data was accepted for the American Society of Clinical Oncology meeting June 2019.

## Supporting information


Data S1

## Data Availability

The data that support the findings of this study are available from the corresponding author upon request.
