# Supplementary material for: A phase 2 and pharmacological study of sapanisertib in patients with relapsed and/or refractory acute lymphoblastic leukemia
Source: Cancer Med. 2023 Nov 13;12(23):21229–39. doi: 10.1002/cam4.6701 (PMC10726920; doi:10.1002/cam4.6701)
Supplement: Supplementary file 1 — Data S1 [file CAM4-12-21229-s001.docx]

**Supplemental Methods**

*Pharmacokinetics*

Blood samples (6 ml, sodium heparin) were obtained during the first cycle of treatment before drug administration on Days 1, 2 and 8; and 0.5, 1, 2, 3, 4, and 8 hours after the oral dose was administered on Day 1 and Day 8. After collection, tubes were immediately cooled in an ice water bath and plasma was isolated by centrifugation (10 minutes; 1,100 - 1,300 x g; 4°C) in a refrigerated centrifuge. Plasma samples were frozen immediately and stored at approximately -70°C or lower until analysis.

Sapanisertib plasma concentrations were measured using a liquid chromatography-mass spectrometry (LC-MS/MS) assay that was validated according to principles outlined in FDA Guidance Documents. Torkinib was used as the internal standard. The LC-MS/MS system consisted of a Xevo G2-XS QTof mass spectrometer (Waters, Milford, MA) equipped with an electrospray ionization (ESI) source coupled to a Waters Acquity I class ultra-performance liquid chromatography (UPLC) system. Data were acquired and analyzed by Waters MassLynx v4.1 software. Sapanisterib and torkinib were separated by reverse-phase chromatography using an InfinityLab Poroshell 120 EC-C18 precolumn (2.1 x 5 mm, 2.7 µm, Agilent, Santa Clara, CA) attached to an InfinityLab Poroshell 120 EC-C18 analytical column (2.1 x 100mm, 2.7 µm Agilent, Santa Clara, CA) under gradient elution. Plasma samples were analyzed after protein precipitation with methanol, filtering through a 0.2 micron filter and dilution with water. Detection of sapanisertib and torkinib was accomplished using the mass spectrometer in positive ESI with analyzer mass mode resolution, with capillary voltage 0.5 kV, source temp 120 °C, desolvation temp 500 °C, cone gas flow 50 L/hr, and desolvation gas flow 800 L/hr. Time of flight (Tof) multiple reaction monitoring (MRM) was used with scan time at 0.10 seconds and data were collected in centroid format using leu-enkephalin as the lockspray reference compound. To monitor ion transitions of 310.14 → 268.1 m/z (cone voltage of 40 V and collision energy of 22 eV) for sapanisertib and 309.15 → 267.11 m/z (cone voltage of 40 V and collision energy of 20 eV) for torkinib.

Plasma concentration-time data were analyzed by standard non-compartmental methods using WinNonlin (Pharsight, Mountain View, CA). Standard descriptive statistics were used to summarize plasma sapanisertib PK parameters. The AUC over the 24-hour dosing interval (τ) on Day 8 was calculated by trapezoidal approximation. Because a 24-hour blood sample was not drawn, the 24-hour plasma concentration after sapanisertib administration was estimated to be equivalent to the pre-dose concentration based on the assumption that steady-state was reached on Day 8. Sapanisertib accumulation was calculated as the ratio of Day 8 AUC_0-24h_: Day 1 AUC_0-24h_. Oral steady-state clearance (Cl_SS_/F) was calculated using the equation, Cl_SS_ = Dose/AUC_τ_.

Pearson’s correlation coefficient (*r*) was used to assess the correlation of group variables (age, gender, body weight, and toxicity) and the clearance of sapanisertib. Statistical significance was defined as *P*<0.05.

*Bone marrow sample isolation*

Cells were isolated from bone marrow or blood samples using Histopaque-1077 (Millipore-Sigma, St. Louis, MO) step gradients. Cells harvested from patients prior to therapy or on Day 8 prior to sapanisertib administration were washed once with ice cold serum-free RPMI 1640 medium containing 10 mM HEPES (pH 7.4 at 21 °C), lysed in buffered 6 M guanidine hydrochloride under reducing conditions, and prepared for SDS-PAGE as previously described ^34^. Cells for *ex vivo* drug exposure were resuspended at a concentration of 1 x 10^6^ cells/ml in RPMI 1640 medium containing 10% (v/v) heat-inactivated fetal bovine serum, 50 U/ml penicillin G, 50 µg/ml streptomycin, and 1 mM glutamine. After incubation for 48 h with the indicated concentration of sapanisertib in the presence of 5 µM Q-VD-OPh, a broad spectrum caspase inhibitor added to inhibit caspase-associated changes in protein expression and phosphorylation, samples were sedimented at 100 x g for 10 minutes, washed once with ice cold serum-free RPMI 1640 medium containing 10 mM HEPES, and lysed in buffered 6 M guanidine hydrochloride under reducing conditions ^34^.

*SDS-polyacrylamide gel electrophoresis (SDS-PAGE) and immunoblotting*

After samples were dialyzed sequentially into 4 M urea and 0.1% (w/v) SDS, lyophilized and reconstituted in SDS-sample buffer consisting of 4 M urea, 2% (w/v) SDS, 62.5 Tris-HCl (pH 6.8 at 4 °C), 10 mM EDTA, and 1% (w/v/) 2-mercaptoethanol, aliquots containing 50 µl of total cellular protein were subjected to SDS-PAGE on gels containing 5-20% polyacrylamide gradients, transferred to nitrocellulose, and probed with antibodies as previously described ^34, 35^. Antibodies against the following antigens were used in these studies: BBC3/PUMA (cat # sc-374223) from Santa Cruz Biotechnology and BCL2L11/BIM (cat #2933S), phospho-Ser^240,241^-S6 (cat #2215S), total S6 (cat #2217S), phospho-Ser-^473^-AKT (cat #9271S), total AKT (cat #9272S), phospho-Thr^37,46^-4EBP1 (cat 9459S), total 4EBP1 (cat #9644S) and GAPDH (cat #2118S) from Cell Signaling Technology (Danvers, MA).

Patients inclusion and appropriateness:

- Acute lymphoblastic leukemia that is either relapsed, refractory to therapy or newly diagnosed ineligibile for for intensive chemotherapy.
- Philadelphia chromosome postive cases were allowed as along as they are not candidate for other therapies against Philadelphia chromosome.
- Patients may receive hydroxyurea or glucosteroids for control of leukocytosis but should be stopped at least 1 day prior to study therapy.
- Age should be 18 years or older
- ECOG performance status should be <3
- Life expectancy of > 2 months
- Patients must have normal organ function as detailed in protocol
- Relapse after stem cell transplant is allowed but with no active GVHD disease.
- Negative serum pregnancy test
- Ability to understand and sign a writen informed consent form
- No prior mTOR inhibitors were allowed (other than GVH prophylaxis)
- HIV+ cases were allowed as long as no history of AIDS defining illness (other conditions apply)
- Patients with diabetes were controlled, evidenced by fasting blood glocuse of <130 mg/kL.
- Patients who were treated with chemotherapy or radiation therapy wihtin 4 weeks were excluded.
- WBC should be <30k prior to start study medicine.
- Patients with known other active cancers (skin cancers were exempted) were excluded
- Patients with allergic reations to compounds similar to sapanisertib were excluded.
- Patients with prolonged QT >480 milliseconds were excluded
- Concurrent use of PPI is not allowed during the study

**Supplemental Table S1**. Response criteria

Complete hematologic response (CR):

< 5% blasts in a non-hypocellular marrow with a granulocyte count of 1 x10^9^/L (or above), and a platelet count of 100 x109/L (or higher) and absence of peripheral blood blasts with complete resolution of any extra medullary disease. The patient is in sustained CR if they have previously achieved a CR and continue to meet the CR criteria (at least 28 days).

CR incomplete (CRi)

Patient meets all CR criteria except for residual neutropenia (ANC<1 x10^9^/L) or thrombocytopenia (platelets<100 x10^9^/L)

Partial Response (PR):

The presence of trilineage hematopoiesis in the bone marrow with recovery of ANC and platelet count to above levels, but with 5-25% bone marrow blasts and ≥50% decrease in bone marrow blast percentage from baseline.

Morphologic leukemia-free state (MLFS):

If bone marrow blasts <5%, absence of Auer rods blasts, absence of extra medullary disease without hematological recovery.

No response (NR):

Failure to achieve CR/CRi/PR/MLFS after 2 cycles of sapanisertib .

Disease Progression:

At least a 25% increase in the absolute number of circulating blasts in peripheral blood, or at least 25% increase in bone marrow blasts, or development of extramedullary disease.

Relapse

Disease recurrence after achieving CR. Disease recurrence is defined by blast ≥ 5% in the bone marrow, or recurrence of peripheral blood blasts > 5% or extramedullary involvement.

**Supplemental Table S2**. All adverse events on study regardless of attribution

|  | | **N** | **%** |
| --- | --- | --- | --- |
| **Patients with at least one:** | **Arm** | 15 | 100 |
| **Grade 3+ Adverse Event** | **A** |  |  |
| **Grade 4+ Adverse Event** | **A** | 13 | 86.7 |
| **Grade 3+ Hem Adverse Event** | **A** | 12 | 80.0 |
| **Grade 4+ Hem Adverse Event** | **A** | 8 | 53.3 |
| **Grade 3+ Non-Hem Adverse Event** | **A** | 13 | 86.7 |
| **Grade 4+ Non-Hem Adverse Event** | **A** | 6 | 40.0 |

| **Adverse Event** | | **Grade** | | | | | | | | | |
| --- | --- | --- | --- | --- | --- | --- | --- | --- | --- | --- | --- |
|  |  | **1** | | **2** | | **3** | | **4** | | **5** | |
|  |  | **N** | **%** | **N** | **%** | **N** | **%** | **N** | **%** | **N** | **%** |
| **Type** | **Arm** |  |  | 2 | 13.3 | 7 | 46.7 |  |  |  |  |
| **Anemia** | **A** |  |  |  |  |  |  |  |  |  |  |
| **Platelet count decreased** | **A** | 1 | 6.7 |  |  |  |  | 6 | 40.0 |  |  |
| **Fatigue** | **A** | 4 | 26.7 | 1 | 6.7 | 1 | 6.7 |  |  |  |  |
| **Anorexia** | **A** | 1 | 6.7 | 3 | 20.0 | 1 | 6.7 |  |  |  |  |
| **Neutrophil count decreased** | **A** | 1 | 6.7 |  |  | 1 | 6.7 | 3 | 20.0 |  |  |
| **Cough** | **A** | 3 | 20.0 | 1 | 6.7 |  |  |  |  |  |  |
| **Epistaxis** | **A** | 3 | 20.0 |  |  | 1 | 6.7 |  |  |  |  |
| **Hyperglycemia** | **A** |  |  | 2 | 13.3 | 2 | 13.3 |  |  |  |  |
| **Lymphocyte count decreased** | **A** |  |  |  |  | 4 | 26.7 |  |  |  |  |
| **Sepsis** | **A** |  |  |  |  |  |  | 4 | 26.7 |  |  |
| **White blood cell decreased** | **A** |  |  | 1 | 6.7 | 1 | 6.7 | 2 | 13.3 |  |  |
| **Abdominal pain** | **A** | 1 | 6.7 | 1 | 6.7 | 1 | 6.7 |  |  |  |  |
| **Alanine aminotransferase increase** | **A** | 2 | 13.3 |  |  | 1 | 6.7 |  |  |  |  |
| **Aspartate aminotransferase increase** | **A** | 2 | 13.3 |  |  | 1 | 6.7 |  |  |  |  |
| **Blood bilirubin increased** | **A** | 1 | 6.7 |  |  | 2 | 13.3 |  |  |  |  |
| **Confusion** | **A** |  |  | 2 | 13.3 | 1 | 6.7 |  |  |  |  |
| **Constipation** | **A** | 3 | 20.0 |  |  |  |  |  |  |  |  |
| **Diarrhea** | **A** | 2 | 13.3 |  |  | 1 | 6.7 |  |  |  |  |
| **Febrile neutropenia** | **A** |  |  |  |  | 3 | 20.0 |  |  |  |  |
| **Headache** | **A** | 1 | 6.7 | 2 | 13.3 |  |  |  |  |  |  |
| **Hypocalcemia** | **A** | 1 | 6.7 | 2 | 13.3 |  |  |  |  |  |  |
| **Hypophosphatemia** | **A** |  |  |  |  | 2 | 13.3 | 1 | 6.7 |  |  |
| **Mucositis oral** | **A** |  |  |  |  | 3 | 20.0 |  |  |  |  |
| **Bruising** | **A** | 2 | 13.3 |  |  |  |  |  |  |  |  |
| **Creatinine increased** | **A** | 1 | 6.7 | 1 | 6.7 |  |  |  |  |  |  |
| **Dry mouth** | **A** | 2 | 13.3 |  |  |  |  |  |  |  |  |
| **Dysphagia** | **A** | 1 | 6.7 | 1 | 6.7 |  |  |  |  |  |  |
| **Edema limbs** | **A** | 2 | 13.3 |  |  |  |  |  |  |  |  |
| **Hypernatremia** | **A** | 1 | 6.7 | 1 | 6.7 |  |  |  |  |  |  |
| **Hypoalbuminemia** | **A** |  |  | 2 | 13.3 |  |  |  |  |  |  |
| **Hyponatremia** | **A** | 2 | 13.3 |  |  |  |  |  |  |  |  |
| **Hypotension** | **A** | 1 | 6.7 | 1 | 6.7 |  |  |  |  |  |  |
| **Lethargy** | **A** |  |  | 2 | 13.3 |  |  |  |  |  |  |
| **Oral pain** | **A** | 1 | 6.7 | 1 | 6.7 |  |  |  |  |  |  |
| **Paresthesia** | **A** | 2 | 13.3 |  |  |  |  |  |  |  |  |
| **Pruritus** | **A** | 1 | 6.7 | 1 | 6.7 |  |  |  |  |  |  |
| **Rash maculo-papular** | **A** | 1 | 6.7 |  |  | 1 | 6.7 |  |  |  |  |
| **Resp, thoracic, mediastinal** | **A** |  |  |  |  | 1 | 6.7 | 1 | 6.7 |  |  |
| **Sinus tachycardia** | **A** | 1 | 6.7 | 1 | 6.7 |  |  |  |  |  |  |
| **Vomiting** | **A** | 2 | 13.3 |  |  |  |  |  |  |  |  |
| **Activated partial thromboplastin time prolonged** | **A** | 1 | 6.7 |  |  |  |  |  |  |  |  |
| **Acute kidney injury** | **A** | 1 | 6.7 |  |  |  |  |  |  |  |  |
| **Alkaline phosphatase increased** | **A** |  |  |  |  |  |  | 1 | 6.7 |  |  |
| **Anal mucositis** | **A** |  |  | 1 | 6.7 |  |  |  |  |  |  |
| **Anxiety** | **A** | 1 | 6.7 |  |  |  |  |  |  |  |  |
| **Ascites** | **A** | 1 | 6.7 |  |  |  |  |  |  |  |  |
| **Atrial fibrillation** | **A** |  |  | 1 | 6.7 |  |  |  |  |  |  |
| **Back pain** | **A** | 1 | 6.7 |  |  |  |  |  |  |  |  |
| **Bladder spasm** | **A** | 1 | 6.7 |  |  |  |  |  |  |  |  |
| **Bloating** | **A** | 1 | 6.7 |  |  |  |  |  |  |  |  |
| **Capillary leak syndrome** | **A** |  |  | 1 | 6.7 |  |  |  |  |  |  |
| **Cardiac disorders** | **A** | 1 | 6.7 |  |  |  |  |  |  |  |  |
| **Chest wall pain** | **A** |  |  | 1 | 6.7 |  |  |  |  |  |  |
| **Cholecystitis** | **A** |  |  | 1 | 6.7 |  |  |  |  |  |  |
| **Death NOS** | **A** |  |  |  |  |  |  |  |  | 1 | 6.7 |
| **Dehydration** | **A** |  |  | 1 | 6.7 |  |  |  |  |  |  |
| **Delirium** | **A** |  |  |  |  | 1 | 6.7 |  |  |  |  |
| **Dizziness** | **A** | 1 | 6.7 |  |  |  |  |  |  |  |  |
| **Dysgeusia** | **A** | 1 | 6.7 |  |  |  |  |  |  |  |  |
| **Dyspnea** | **A** | 1 | 6.7 |  |  |  |  |  |  |  |  |
| **Ear pain** | **A** | 1 | 6.7 |  |  |  |  |  |  |  |  |
| **Esophagitis** | **A** |  |  | 1 | 6.7 |  |  |  |  |  |  |
| **Facial pain** | **A** |  |  | 1 | 6.7 |  |  |  |  |  |  |
| **Fall** | **A** |  |  | 1 | 6.7 |  |  |  |  |  |  |
| **Fever** | **A** | 1 | 6.7 |  |  |  |  |  |  |  |  |
| **Fibrinogen decreased** | **A** |  |  |  |  | 1 | 6.7 |  |  |  |  |
| **Gait disturbance** | **A** | 1 | 6.7 |  |  |  |  |  |  |  |  |
| **Generalized muscle weakness** | **A** |  |  |  |  | 1 | 6.7 |  |  |  |  |
| **Hematuria** | **A** | 1 | 6.7 |  |  |  |  |  |  |  |  |
| **Hepatic failure** | **A** |  |  |  |  | 1 | 6.7 |  |  |  |  |
| **Hyperhidrosis** | **A** | 1 | 6.7 |  |  |  |  |  |  |  |  |
| **Hypermagnesemia** | **A** | 1 | 6.7 |  |  |  |  |  |  |  |  |
| **Hypertension** | **A** |  |  | 1 | 6.7 |  |  |  |  |  |  |
| **Hypertriglyceridemia** | **A** | 1 | 6.7 |  |  |  |  |  |  |  |  |
| **Hypokalemia** | **A** | 1 | 6.7 |  |  |  |  |  |  |  |  |
| **INR increased** | **A** | 1 | 6.7 |  |  |  |  |  |  |  |  |
| **Infections and infestations** | **A** |  |  | 1 | 6.7 |  |  |  |  |  |  |
| **Insomnia** | **A** | 1 | 6.7 |  |  |  |  |  |  |  |  |
| **Intracranial hemorrhage** | **A** |  |  |  |  | 1 | 6.7 |  |  |  |  |
| **Leukocytosis** | **A** |  |  |  |  | 1 | 6.7 |  |  |  |  |
| **Lung infection** | **A** |  |  |  |  | 1 | 6.7 |  |  |  |  |
| **Nausea** | **A** | 1 | 6.7 |  |  |  |  |  |  |  |  |
| **Neck pain** | **A** | 1 | 6.7 |  |  |  |  |  |  |  |  |
| **Neoplasms benign, malignancy** | **A** |  |  |  |  |  |  |  |  | 1 | 6.7 |
| **Non-cardiac chest pain** | **A** |  |  | 1 | 6.7 |  |  |  |  |  |  |
| **Pain** | **A** | 1 | 6.7 |  |  |  |  |  |  |  |  |
| **Pain in extremity** | **A** |  |  |  |  | 1 | 6.7 |  |  |  |  |
| **Pharyngolaryngeal pain** | **A** |  |  | 1 | 6.7 |  |  |  |  |  |  |
| **Pleural effusion** | **A** | 1 | 6.7 |  |  |  |  |  |  |  |  |
| **Pneumonitis** | **A** |  |  |  |  | 1 | 6.7 |  |  |  |  |
| **Proteinuria** | **A** | 1 | 6.7 |  |  |  |  |  |  |  |  |
| **Pulmonary edema** | **A** | 1 | 6.7 |  |  |  |  |  |  |  |  |
| **Seizure** | **A** |  |  |  |  | 1 | 6.7 |  |  |  |  |
| **Sinus bradycardia** | **A** | 1 | 6.7 |  |  |  |  |  |  |  |  |
| **Skin hyperpigmentation** | **A** | 1 | 6.7 |  |  |  |  |  |  |  |  |
| **Sore throat** | **A** | 1 | 6.7 |  |  |  |  |  |  |  |  |
| **Supraventricular tachycardia** | **A** |  |  | 1 | 6.7 |  |  |  |  |  |  |
| **Tremor** | **A** |  |  | 1 | 6.7 |  |  |  |  |  |  |
| **Tumor lysis syndrome** | **A** |  |  |  |  | 1 | 6.7 |  |  |  |  |
| **Urinary incontinence** | **A** |  |  | 1 | 6.7 |  |  |  |  |  |  |
| **Urinary retention** | **A** |  |  | 1 | 6.7 |  |  |  |  |  |  |
| **Urinary tract infection** | **A** |  |  | 1 | 6.7 |  |  |  |  |  |  |
| **Urinary urgency** | **A** |  |  | 1 | 6.7 |  |  |  |  |  |  |
| **Vaginal inflammation** | **A** |  |  | 1 | 6.7 |  |  |  |  |  |  |
| **Weight loss** | **A** | 1 | 6.7 |  |  |  |  |  |  |  |  |
| **Wheezing** | **A** |  |  | 1 | 6.7 |  |  |  |  |  |  |

**Supplemental Table S3**. Select adverse events possibly related to Sapanisertib based on cell-lineage (B- vs T-cell)

| **Listing of Grade 3+ Adverse Events Max Grade per Patient Per Event Number of Evaluable Patients Arm: B-Cell=10   T-Cell=5** | | | | |
| --- | --- | --- | --- | --- |
|  |  | **Grade of Adverse Event** | | |
|  | **Arm** | **3-Severe** | **4-LifeThr** | **5-Lethal** |
|  | | **N (%)** | **N (%)** | **N (%)** |
| **Hematologic Adverse Events** | | | | |
| **Blood/Bone Marrow** | | | | |
| Anemia | B-Cell | 1 (10%) | 0 (0%) | 0 (0%) |
|  | T-Cell | 0 (0%) | 0 (0%) | 0 (0%) |
| Leukocytosis | B-Cell | 0 (0%) | 0 (0%) | 0 (0%) |
|  | T-Cell | 1 (20%) | 0 (0%) | 0 (0%) |
| Lymphocyte count decreased | B-Cell | 2 (20%) | 0 (0%) | 0 (0%) |
|  | T-Cell | 0 (0%) | 0 (0%) | 0 (0%) |
| Neutrophil count decreased | B-Cell | 0 (0%) | 1 (10%) | 0 (0%) |
|  | T-Cell | 0 (0%) | 0 (0%) | 0 (0%) |
| Platelet count decreased | B-Cell | 0 (0%) | 1 (10%) | 0 (0%) |
|  | T-Cell | 2 (40%) | 1 (20%) | 0 (0%) |
| White blood cell decreased | B-Cell | 0 (0%) | 2 (20%) | 0 (0%) |
|  | T-Cell | 0 (0%) | 0 (0%) | 0 (0%) |
| **Non-Hematologic Adverse Events** | | | | |
| **Blood and lymphatic sys disord** | | | | |
| Febrile neutropenia | B-Cell | 1 (10%) | 0 (0%) | 0 (0%) |
|  | T-Cell | 0 (0%) | 0 (0%) | 0 (0%) |
| **Gastrointestinal disorders** | | | | |
| Abdominal pain | B-Cell | 0 (0%) | 0 (0%) | 0 (0%) |
|  | T-Cell | 1 (20%) | 0 (0%) | 0 (0%) |
| Mucositis oral | B-Cell | 3 (30%) | 0 (0%) | 0 (0%) |
|  | T-Cell | 0 (0%) | 0 (0%) | 0 (0%) |
| **Gen disord and admin site cond** | | | | |
| Fatigue | B-Cell | 1 (10%) | 0 (0%) | 0 (0%) |
|  | T-Cell | 0 (0%) | 0 (0%) | 0 (0%) |
| **Metabol and nutrition disord** | | | | |
| Anorexia | B-Cell | 1 (10%) | 0 (0%) | 0 (0%) |
|  | T-Cell | 0 (0%) | 0 (0%) | 0 (0%) |
| Hyperglycemia | B-Cell | 2 (20%) | 0 (0%) | 0 (0%) |
|  | T-Cell | 0 (0%) | 0 (0%) | 0 (0%) |
| **Psychiatric disorders** | | | | |
| Confusion | B-Cell | 1 (10%) | 0 (0%) | 0 (0%) |
|  | T-Cell | 0 (0%) | 0 (0%) | 0 (0%) |
| **Respirat, thor, mediast disord** | | | | |
| Pneumonitis | B-Cell | 0 (0%) | 0 (0%) | 0 (0%) |
|  | T-Cell | 1 (20%) | 0 (0%) | 0 (0%) |
| Resp, thoracic, mediastinal - Oth spec | B-Cell | 0 (0%) | 1 (10%) | 0 (0%) |
|  | T-Cell | 0 (0%) | 0 (0%) | 0 (0%) |
| **UNK** | | | | |
| Neoplasms benign, mal, uncpec - Oth spec | B-Cell | 0 (0%) | 0 (0%) | 0 (0%) |
|  | T-Cell | 1 (20%) | 0 (0%) | 0 (0%) |


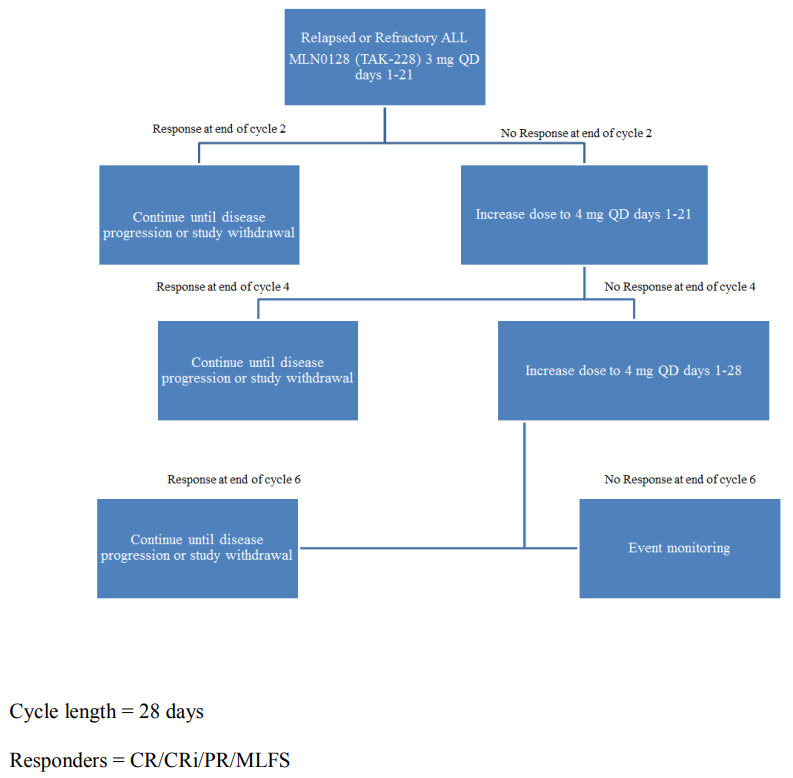


**Supplemental Figure S1**. Schema of NCI-9775 trial


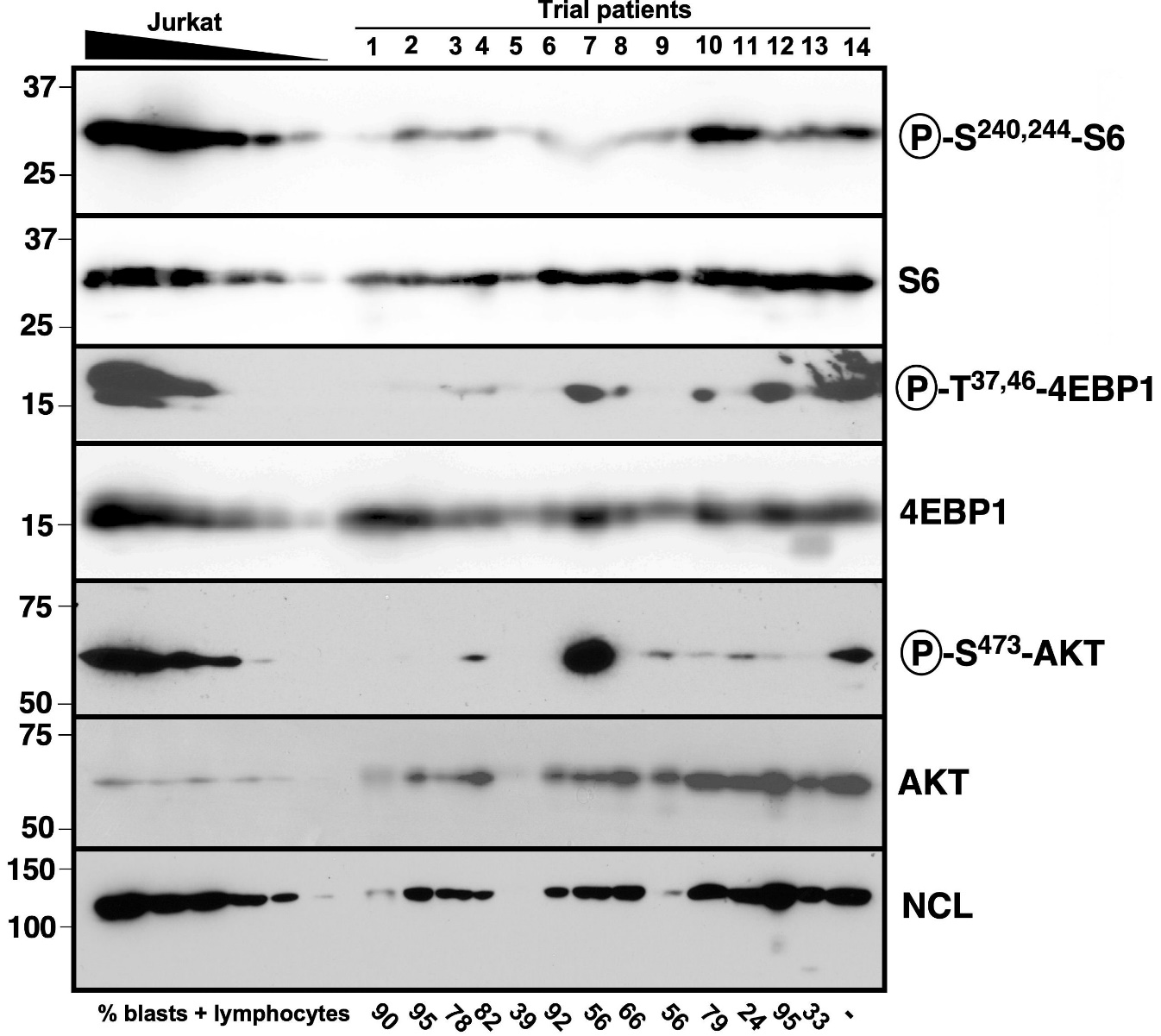


**Supplemental Figure S2. Pretreatment levels of mTOR-associated RPS6, 4EBP1 and AKT phosphorylation.** Whole cell lysates prepared from 5 x 10^5^ marrow mononuclear cells from each of 14 patients enrolled on this study were subjected to SDS-polyacrylamide gel electrophoresis, transferred to nitrocellulose, and probed with antibodies to the indicated antigens. A series of serial 2-fold dilutions of whole cell lysate from the T-cell ALL line Jurkat (starting with lysate from 5 x 10^5^ cells) provided an indication of how much lower the mTOR-mediated phosphorylations were in the clinical samples compared to a continuously cycling acute leukemia cell line. The housekeeping protein nucleolin (NCL) served as a loading control.
